# Biodegradation of petroleum tar in contaminated sediments of the Eastern Mediterranean shores and associated microbial dynamics

**DOI:** 10.1128/aem.00258-25

**Published:** 2025-06-12

**Authors:** Baraa Al Haj Chehadeh, Farah Ali Ahmad, Darine A. Salam

**Affiliations:** 1 Department of Civil and Environmental Engineering, Maroun Semaan Faculty of Engineering and Architecture, American University of Beirut 538704, Beirut, Lebanon; University of Delaware, Lewes, Delaware, USA

**Keywords:** Mediterranean coast, marine sediments, tar residues, biodegradation, microbial community evolution

## Abstract

The Eastern Mediterranean coasts were impacted by several oil spills over the past two decades where tar residues were encountered on the shoreline. The majority of research on marine tar residues has focused on tar formation, chemical composition, transport, and fingerprinting to trace petroleum oil spills. Only a few studies have assessed the biodegradation of tar in the marine environment, and no studies have examined the evolution of the associated microbial communities. This research evaluated the biodegradation of tar residues in contaminated beach sediments of the Eastern Mediterranean coast of Lebanon. Biodegradation experiments were conducted in laboratory microcosms over a period of 56 days at 18°C and 28°C. Tar removal throughout the incubation period was monitored by the measurement of residual *n*-alkanes and polycyclic aromatic hydrocarbons (PAHs) using gas chromatography-mass spectrometry. Additionally, the diversity and evolution of the microbial community structure throughout the tar biodegradation experiments were determined using 16S rRNA gene sequencing. The biodegradation rates of total alkanes and total PAHs were 0.035 day^−1^ and 0.023 day^−1^, respectively, at 18°C, and increased to 0.110 day^−1^ and 0.055 day^−1^ at 28°C. Microbial analysis revealed a shift in the microbial community from generalist hydrocarbon degraders at the beginning of the biodegradation process to more specialized groups as the experiments progressed. Key genera involved in tar biodegradation included Erythrobacter, Bacillus, Alcanivorax, Marinobacter, Actinomarinales, and Pseudomonas. These findings highlight the potential of the Eastern Mediterranean coast to naturally degrade tar contamination and enhance our understanding of the microbial dynamics associated with tar biodegradation.

**IMPORTANCE:**

The planned oil and gas extraction activities of the Eastern Mediterranean coasts increase the risk of potential oil spills and threaten the Mediterranean shoreline with devastating impacts. A recent oil spill has resulted in huge amounts of tar residues washing up along the Lebanese southern coastline, affecting Nature Reserve shores known to be a nesting ground for several species of endangered turtles. The majority of research conducted on marine tar residues has studied tar formation, distribution and prevalence, chemical composition and tracing, transport mechanisms, as well as human and ecological effects. The biodegradation of spilled petroleum tar in aquatic media and the associated microbial dynamics are still poorly addressed in the literature. This study contributes to the state of knowledge and current scarce literature on petroleum tar biodegradation in marine environments and provides guidelines to spill responders for an effective bioremediation response plan to address future potential tar contamination.

## INTRODUCTION

The Eastern Mediterranean coasts were impacted by several oil spills over the past two decades where tar residues were encountered on the shoreline. In July 2006, 150 km of Lebanese and Syrian coastline were polluted following an attack on the Jiyeh thermal power plant south of Beirut, Lebanon, which resulted in the spill of an estimated 10,000–15,000 tons of medium/heavy fuel oil (1, 2). Some of the oil had emulsified and solidified along the Lebanese shore, clinging to sand and rocks, while the oil that spilled into the water was more fluid, later drying to a tarry residue that was deposited on beaches (3). The region is also susceptible to oil leakage from oil refineries and tanks that can cause long-term negative effects on coastal ecosystems and communities. In August 2021, a tank at the Baniyas thermal station in Syria ruptured, resulting in an oil spill of 15,000 tons into the Mediterranean Sea (4). The spilled oil covered an area of 800 km^2^ and threatened the northern shores of Turkey and Cyprus (4). Another notable oil spill occurred in February 2021 along the northern Israeli coast from an unknown oil tanker. The spill resulted in huge amounts of tar and oil residues washing up along the Lebanese southern coastline (5). The resulting oil pollution has covered around 40% of the Nature Reserve shores in Tyre, a protected environment rich in biodiversity and known to be a nesting ground for several endangered species of turtles (5). At least five loggerhead turtles died following the oil spill (6). Tar balls of variable sizes and as small as 5 mm were found scattered on several other beaches along the southern Lebanese coastline. With the anticipated oil and gas extraction off the Eastern Mediterranean coasts, including the Lebanese coastline, risks of future oil spills and pollution with tar residues are becoming a major concern. Consequently, an understanding of the fate of tar residues is essential to define proper mitigation measures for tar pollution and protect the marine ecosystem.

Petroleum tar is essentially a semi-solid residue formed following the physical, chemical, and biological weathering of spilled oil (7). The composition and behavior of the spilled tar are impacted by these combined weathering processes, as well as the original oil composition and the time elapsed after the spill (8). Petroleum tar samples typically have lower amounts of lower molecular weight hydrocarbons and higher amounts of higher molecular weight hydrocarbons compared to petroleum oil, which can be attributed to the different weathering processes that these samples undergo in the marine environment, including evaporation and dissolution. Analysis of tar samples reported in the literature showed that total alkanes range from 0.6 to 192 mg/g of tar, while total polycyclic aromatic hydrocarbons (PAHs) range from 6 to 557 µg/g (9). Chromatograms of tar samples are usually characterized by scarce amounts of compounds with low retention times (less than 10 minutes) and a notable feature resembling a “hump,” which corresponds to the unresolved complex mixture, typically attributed to the low maturity of the tar sample or byproducts of the weathering of the sample (10, 11). The chromatogram of tar samples collected from Tyre Beach in Southern Lebanon following the August 2021 spill is shown in the supplemental material.

Marine tar residues can remain buried in the sea floor, decompose, or be transported ashore via currents and waves (7). Long-term studies conducted following oil spill incidents have shown that tar residues persist remarkably in the environment, with biodegradation being chiefly responsible for their ultimate removal (7). The majority of research conducted on marine tar residues has studied the tar formation, distribution, transport, composition, and impacts, in addition to tracing petroleum oil spills (9, 12–16). Only a few studies have evaluated the biodegradation of tar residues in the marine environment. A recent study (17) assessed the biodegradation of petroleum tar by 12 associated bacterial consortia for the remediation of Goa State Beaches in India. The results indicated a depletion of 53.69%–97.78% in *n*-alkanes and 22.78%–61.98% in PAHs by different consortia, with *Pseudomonas* and *Alcanivorax* species contributing to maximum biodegradation rates. Another study (18) demonstrated the potential for bioremediation of sites polluted with petroleum tar in Assam, India, by *Pseudomonas* species. The biodegradation of coal tar, having a similar PAH composition to that of petroleum tar, by indigenous bacterial communities found in soil was also reported in other studies (19, 20). Tar hydrocarbon biodegradation studies have reported the biodegradation of *n*-alkanes and PAHs, which were amenable to degradation by microbial communities during the studied incubation periods, as opposed to the other more complex and recalcitrant compounds, such as resins and asphaltenes.

The scarcity of studies on the biodegradation of marine tar makes it poorly understood. Reported biodegradation rates of tar have proven to be highly variable depending on different factors. Namely, the source of spilled oil and its chemical composition, as well as the availability of specific microbes, influence the susceptibility of the tar residues to biodegradation (8). In addition, among other environmental variables, variations in temperature highly influence tar biodegradation rates, as temperature impacts the physical properties of the oil residue (density, viscosity, surface tension, etc.) as well as the microbial activity (8).

This study assesses the biodegradation of petroleum tar in contaminated marine sediments collected from the Eastern Mediterranean Shores of Lebanon and characterizes the different microbial communities involved during the biodegradation process. The impact of temperature on the tar biodegradation kinetics and microbial structure evolution was also investigated. The results of this study contribute to the current scarce knowledge on petroleum tar biodegradation in marine environments and permit the determination of microbial dynamics during the tar biodegradation process.

## MATERIALS AND METHODS

### Sediment sample collection and characterization

Sediment samples used in the tar biodegradation experiments were manually collected from the beach of Tyre, Southern Lebanon, which was the most affected by the February 2021 tar pollution. This ensured the collection of sediments enriched with petroleum hydrocarbon degraders. The sediments were collected from the beach surface at water depths in the range of 30–50 cm and sieved using a 2 mm pore-size sieve. The water depths at which the sediment samples were collected represent the intertidal zones, which are the areas of the foreshore and seabed that are submerged at high tide and exposed to the air at low tide, making them subject to aerobic conditions. Collected sediments were analyzed in triplicates for background concentrations of nitrogen and phosphorus necessary for microbial activity and exhibited a total nitrogen concentration of 0.57 mg/Kg ± 0.02 and total phosphorus content below the detection limit. The sediments were also analyzed for hydrocarbon-degrading bacteria using the most probable number according to the procedure outlined in (21). Light Arabian petroleum crude oil was added as a carbon source. The analysis demonstrated the existence of hydrocarbon-degrading microbial communities making the sediments intrinsically capable of mitigating petroleum oil contamination, with a reported bacterial concentration of 13,630 /mL. Detailed results and discussion of the chemical and bacteriological characterization of the sediment samples are shown in the supplemental material.

### Tar biodegradation experiments

#### Microcosm preparation and sampling

Petroleum tar from the beach of Tyre, South Lebanon, was acquired from Greenpeace, a nongovernmental organization that participated in cleanup efforts following the occurrence of the 2021 oil spill and tar pollution of the Lebanese southern coastline. Biodegradation experiments of tar in synthetically contaminated beach sediments were carried out in 250 mL flasks including 100 g (wet weight) of sieved beach sediments. A contamination dose of 0.7 g of tar/Kg of beach sediment was selected for the experiments, as it is consistent with levels of petroleum oil spills reported in marine ecosystems (22, 23). The highly viscous and sticky nature of tar made it difficult to manipulate and hampered weighing and adding exact amounts of the contaminant into the microcosms. For this reason, an aliquot of the tar sample was initially dissolved in dichloromethane (DCM). A minimal volume of the tar solution (0.82 mL) was then spiked into each of the microcosms to achieve a tar contamination level of 0.7 g/Kg of beach sediment. The microcosms were then left to equilibrate for 2 hours to allow for the evaporation of the DCM and the distribution of the tar throughout the microcosms. While monitoring the equilibration, it was observed that the minimal amount of used DCM evaporated rapidly, leaving the resolidified tar homogeneously mixed with the sediments. Therefore, the structural integrity of the tar was maintained, and microbial accessibility was not artificially enhanced by solvent disruption. A minimal volume of distilled water (~10 mL) was added to the matrix and replenished during the experiments to facilitate shaking at 200 rpm. Because background levels of phosphorus and nitrogen were not adequate to sustain microbial activity, beach sediments were initially supplemented with these nutrients to achieve a C:N:P ratio of 100:5:1 (22, 24) as explained in the supplemental material. The indigenous microbial community found in the beach sediments was relied upon for biodegradation, without the addition of any exogenous cultures. As the primary goal of the experiments was to assess the biodegradability of tar by the existing microbial communities in the sediments, biostimulation through the addition of N and P ensures that biodegradation would not be limited by nutrient deficiency, allowing for a more accurate evaluation of microbial activity and evolution throughout tar biodegradation.

Biodegradation experiments lasted for 56 days and were conducted at 18°C and 28°C, characteristic of average seawater temperatures experienced during the cold and warm seasons in Lebanon. For each treatment, a total of 13 sampling events were performed over the incubation period corresponding to days 0, 2, 4, 8, 11, 14, 17, 21, 28, 35, 42, 49, and 56 of the experiments. At each sampling event, triplicate biotic samples were sacrificed for a total of 39 samples per treatment. In addition to the biotic microcosms, three abiotic blanks consisting of the autoclaved matrix and added tar were also prepared per treatment to account for abiotic losses of the tar in the absence of any microbial activity. Sodium azide (NaN_3_; 500 mg/Kg) was added to the blanks as a sterilant to prevent microbial contamination during the incubation period of the microcosms. Abiotic samples were sacrificed during the last sampling event on day 56.

Extraction of the residual tar from the sacrificed microcosms was conducted using an accelerated solvent extractor (Thermo Scientific Dionex, ASE 350) apparatus following the method outlined by Richter (25). Liquid-liquid extraction was then conducted to remove the water from the extracts using DCM as the solvent, and the decantates comprising the organic fraction were passed through magnesium sulfate for further drying. The resulting dry extracts were concentrated to 1–2 mL under rotary evaporation and were then brought to a 25 mL volume by adding DCM in a 25 mL volumetric flask.

#### Tar biodegradation rates

To monitor the biodegradation of tar throughout the experiments, concentrations of the targeted *n-*alkanes and PAHs in the sacrificed microcosms were measured using gas chromatography–mass spectrometry (GC-MS) based on an internal standard method. Measured concentrations were normalized to hopane to account for potential losses that might result from physical factors acting on tar constituents and ultimately isolate the effect of biodegradation (21, 22). The normalized concentrations of the individual target alkanes were added to obtain the concentration of total alkanes in each sample. The mean of each triplicate sample was calculated to obtain the data point corresponding to each sampling event at each temperature. The concentration of total PAHs at each sampling event was calculated similarly. To calculate the biodegradation rate coefficients of both individual and total alkanes and PAHs, the obtained data were fitted into a first-order model using nonlinear regression:


(1) $$C_{t}=C_{0}exp^{-kt}$$

*C*_0_ is the hopane-normalized initial concentration of petroleum hydrocarbons, *C*_*t*_ is the hopane-normalized concentration of the analytes at time *t* (days), and *k* corresponds to the first-order biodegradation rate coefficient (day^−1^). The analysis was performed on each individual alkane and PAH to obtain the individual biodegradation rate coefficients, as well as on total alkanes and PAHs to obtain their overall biodegradation rate coefficients.

### Microbial community characterization

To study the diversity and evolution of the microbial populations involved in tar biodegradation, the microbial communities occurring during the biodegradation process were characterized using 16S rRNA gene sequencing for the sampling events corresponding to days 0, 2, 4, 8, 11, 14, 17, 21, 28, 42, and 56 of the experiments. At each sampling event, aliquots of sediments were collected from the sacrificed microcosms, and DNA extraction was conducted using the DNeasy PowerSoil Kit (Qiagen, USA). Resulting DNA extracts from triplicate microcosms sacrificed at a specific sampling event and temperature condition were combined in one sample. This resulted in a total of 22 DNA samples from both treatments. The extracted DNA samples were shipped to Molecular Research, LP MR DNA (Shallowater, TX, USA), for the characterization of the microbial communities. Details on DNA amplification, sequencing, and sequence data processing performed at MR DNA are provided in the supplemental material.

R statistical language (version 4.2.2 - 2022) was used to analyze and visualize the zero-radius operational taxonomic units (zOTUs) obtained for the different samples using the ampvis2 R package, with Rstudio as the integrated development environment. In addition to plotting heatmaps showing the evolution of the microbial community throughout the experiments, a nonmetric multidimensional scaling (NMDS) plot was generated using microbial community composition data, specifically at the genus level, based on the Bray-Curtis dissimilarity to capture and visualize the differences in community composition across the samples of the two experiments. In addition, Statistical Analysis of Metagenomic Profiles software was used to evaluate the statistical differences between microbial populations among the two temperature treatments. For this aim, Welch’s two-sided *t*-test was applied as the statistical test with a confidence interval of 95%.

## RESULTS AND DISCUSSION

### Biodegradation of tar

#### Biodegradation kinetics

**Fig 1:**
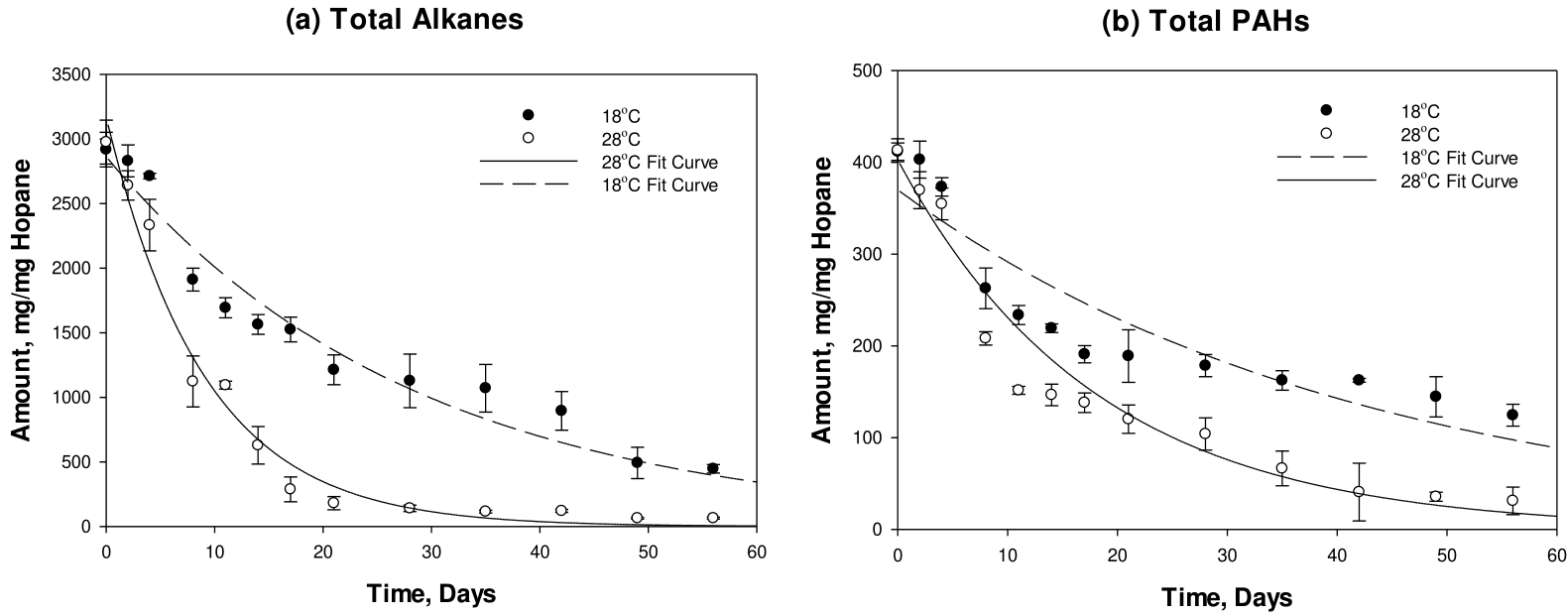
Biodegradation kinetics of total alkanes (**a**) and total PAHs (**b**) in beach sediment samples at 18⁰C and 28⁰C. Data points represent average values of independent triplicates. The units for the error bars are displayed as ±1 SEM.

Figure 1 presents the biodegradation curves of total alkanes and PAHs at 18°C and 28°C.

Total alkanes exhibited a more rapid depletion at 28°C, with a major drop in concentration occurring between days 4 and 8, while a more gradual decrease in concentration was observed at 18°C. At 28°C, the biodegradation curve plateaued starting day 21, with almost complete depletion of total alkanes being achieved at the end of the experiment. In contrast, alkanes degradation leveled off at day 49 at 18°C with a residual alkanes concentration of around 500 mg/mg of hopane measured toward the end of the incubation period. The measured first-order degradation rate of alkanes at 28°C was 0.1106 day^−1^ and was almost triple the rate attained at 18°C of 0.0353 day^−1^. The depletion of total alkanes over the 56-day incubation period amounted to 84.71% and 97.81% at 18°C and 28°C, respectively. Similarly, total PAHs underwent a more rapid degradation at 28°C, with a measured biodegradation rate of 0.0556 day^−1^, double the rate at 18°C of 0.0238 day^−1^. Degradation at both temperatures was gradual and plateaued starting day 49. Almost complete depletion of total PAHs was observed at 28°C, while the reported concentration at the end of the 18°C biodegradation experiment was 114.4 mg/mg of hopane. The achieved removal of total PAHs over the 56-day incubation period was 72.21% and 92.47% at 18°C and 28°C, respectively. Table 1 presents the first-order biodegradation rate coefficients of total alkanes and PAHs in tar and the corresponding temperature coefficients for the two treatments.

**TABLE 1:** Biodegradation rates of total alkanes and PAHs^*a*^

*T* (°C)	Total *n*-alkanes				Total PAHs			
	*k*	SEM	*R* ^2^	*k*_28_/*k*_18_	*k*	SEM	*R* ^2^	*k*_28_/*k*_18_
28	0.1106	0.0083	0.9786	3.133	0.0556	0.0051	0.9559	2.336
18	0.0353	0.0030	0.9494		0.0238	0.0041	0.7715	

^*a*^ *R*^2^, correlation coefficient; *k*_28_/*k*_18_ temperature coefficients for the two treatments.

Measured temperature coefficients for a difference of 10°C were 3.133 for total alkanes and 2.336 for total PAHs, implying typical biodegradation behavior. An increase of 10°C generally results in doubling the biodegradation rate (8). This is expected as microbial activity is enhanced at higher temperatures. In addition to affecting the rate of biodegradation, temperature variation also impacts the physio-chemical properties of petroleum tar. Increased temperatures decrease the surface tension and viscosity of tar and increase the solubility of low molecular weight (LMW) alkanes and PAHs, thereby enhancing the tar’s susceptibility to biodegradation (8, 26). The effect of increasing the temperature was more pronounced on alkanes than PAHs due to the more complex structure of PAHs, which makes them less readily available for biodegradation than alkanes (8).

The relatively high removal rates of total alkanes and total PAHs achieved at both temperatures are indicative of the occurrence of hydrocarbon degraders of tar components in the contaminated beach sediments. A study on the degradation of tar by 12 different bacterial consortia isolated from tarballs in the Goa State of India reported removal ranges of total alkanes and total PAHs of 53.69%–97.78% and 22.78%–61.98%, respectively, over a period of 45 days at 22°C (27). The study identified that bacterial consortia consisting of several bacterial strains were more effective in the degradation of tar than individual bacterial strains. In another study (19), the biodegradation of coal tar PAHs was monitored under different nutrient amendments and biosurfactant treatments for a period of 55 days at 20°C. In this case, PAH removal ranged between 20% and 70%. In this study, it is expected that beach sediments used in the biodegradation experiments comprise hydrocarbon degraders contributing to the relatively high measured removal rates of alkanes and PAHs. Indeed, the previous exposure of the sediment to tar pollution would have enriched the indigenous microbial communities with degraders of the tar components. Furthermore, the microbial characterization of the shoreline of Lebanon (28) showed the occurrence of hydrocarbon-degrading taxa. In a more recent study, the authors assessed the spatio-temporal variation of the microbial community on the Lebanese coast and reported a notable change in the microbial population found in sediment samples toward hydrocarbon degraders, due to the presence of petroleum hydrocarbon pollution (29).

It is important to note that measured tar biodegradation rates in this study were lower than the biodegradation rates of Arabian crude petroleum oil reported in a previous study (22) under the same experimental conditions. Biodegradation rates of total alkanes (0.114 day^−1^ at 18°C and 0.244 day^−1^ at 28°C) and total PAHs (0.057 day^−1^ at 18°C and 0.109 day^−1^ at 28°C) in the used crude oil were at least double those reported in tar biodegradation in this study. This is attributed to the small surface area of the tar relative to its volume, which hampers the biodegradation process, as well as to the dominance of the higher molecular weight alkanes and PAHs in weathered tar.

#### Biodegradation of individual alkanes and PAHs

**Fig 2:**
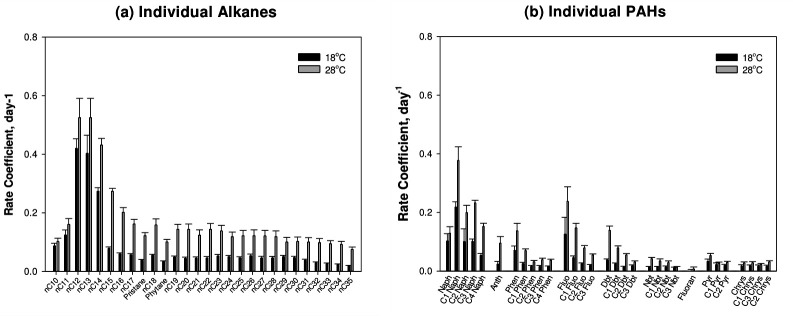
Biodegradation rate coefficients of individual (**a**) alkanes and (**b**) PAHs. Data points represent average values of independent triplicates. The units for the error bars are displayed as ±1 SEM. PAHs include naphthalene (Naph), phenanthrene (Phen), fluorene (Fluo), dibenzothiophene (Dbt), naphthobenzothiophene (Nbt), pyrene (Pyr), chrysene (Chrys), and their alkylated homologs, as well as anthracene (Anth) and fluoranthene (Fluo).

The biodegradation rate coefficients of alkanes and PAHs at the two studied temperatures are presented in Fig. 2.

Biodegradation rate coefficients of alkanes decreased with increasing length of the carbon chain. This is consistent with the higher susceptibility of smaller hydrocarbon chains to microbial degradation (30, 31). However, the lower carbon number alkanes nC10 and nC11 exhibited relatively lower biodegradation rates at both temperatures. This is attributed to their relatively reduced occurrence in the weathered tar, due to their higher volatility and biodegradability, resulting thus in reduced biodegradation rates. Furthermore, the results show lower biodegradation rates of isoprenoid hydrocarbons (pristane and phytane) compared to their straight-chain counterparts. Isoprenoids are branched-chain saturated hydrocarbons more resistant to biodegradation than straight-chain alkanes (11, 32).

Similarly, the general trend in the degradation of PAHs depicts a decrease in biodegradation rates with an increased number of alkyl groups and carbon rings, which is consistent with typical biodegradation behavior (30). However, an exception to this trend is observed with naphthalene, which shows a lower removal rate than its alkylated homologs, namely at 28°C. This can be attributed to the high volatility of naphthalene leading to initial low amounts of the aromatic compound in tar. Higher biodegradation rates were observed for three-ring PAHs and their alkylated counterparts (anthracene, C_0–4_ phenanthrene, C_0–3_ fluorene, and C_0–3_ dibenzothiophene) compared to the higher carbon ring PAHs. In a study assessing the biodegradation of petroleum alkanes and PAHs by a strain of *Pseudomonas*, higher biodegradation rates were reported for fluorene and phenanthrene compared to pyrene. The authors reported that the increase in the number of rings leads to increased hydrophobicity and decreased solubility, which in turn reduces the availability of PAHs for microbial degradation (33). It is worth noting the higher degradation rates of fluorene as compared to phenanthrene and anthracene with the same number of carbon rings. Indeed, fluorene, having only two benzene rings and a five-membered middle ring with no aromatic properties, is reported to be more readily degradable than all other PAHs, including phenanthrene and anthracene, which have three fused benzene rings.

Excluding compounds with high reported volatility, measured biodegradation rates of individual alkanes and PAHs at 28°C were typically double the rates reported at 18°C. Only pyrene and chrysene, along with their alkylated homologs, exhibited close biodegradation rates at both temperatures due to their resistant nature to microbial degradation. It is important to note that fluoranthene showed extremely low initial concentrations in tar, which prevented the proper evaluation of its biodegradation.

### Microbial community characterization and evolution

#### Microbial diversity

The total number of sequencing reads in the analyzed samples ranged from 21,325 to 31,966, with a total number of zOTUs of 1,211. The Shannon alpha diversity index (Table S2 in the supplemental material) showed high values ranging from 2.67 to 5.73, indicating high diversity in the microbial community for both biodegradation treatments. Interestingly, a general decreasing trend was observed in the Shannon index with the advancement in sampling events, indicating a decrease in diversity as biodegradation progressed.

**Fig 3:**
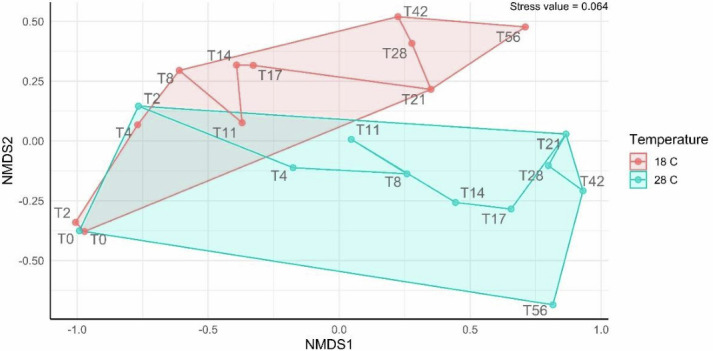
NMDS plot showing the dissimilarity of microbial populations at the two tested temperatures. The points T0, T2, T4, T8, T11, T14, T17, T21, T28, T42, and T56 represent the sampling events on days 0, 2, 4, 8, 11, 14, 17, 21, 28, 42, and 56, respectively.

To assess relationships among microbial operational taxonomic units, an NMDS plot was used (Fig. 3) and displayed clear clustering of the microbial communities in two groups, corresponding to the microbial populations characterized at the two tested temperature conditions. This indicates a dissimilarity in microbial populations. The points corresponding to the sampling events on day 0 showed very similar microbial composition for both treatments since the same beach sediments collected from the same site at the same time were used in both experiments. Subsequently, the microbial community at 28°C presented a faster shift during the first few sampling events, as there appears to be a considerable distance between points T0, T2, and T4. On the other hand, the microbial community at 18°C exhibited a slower shift from the background community at T0, as point T2 appears to be very close to T0. This can be attributed to enhanced microbial metabolism at the higher temperature, which permits faster biodegradation rates of hydrocarbons and contributes thus to a faster appearance of the consecutive microbial populations associated with the biodegradation of specific residual hydrocarbons at each stage of the biodegradation process (8, 26).

#### Microbial structure and evolution

**Fig 4:**
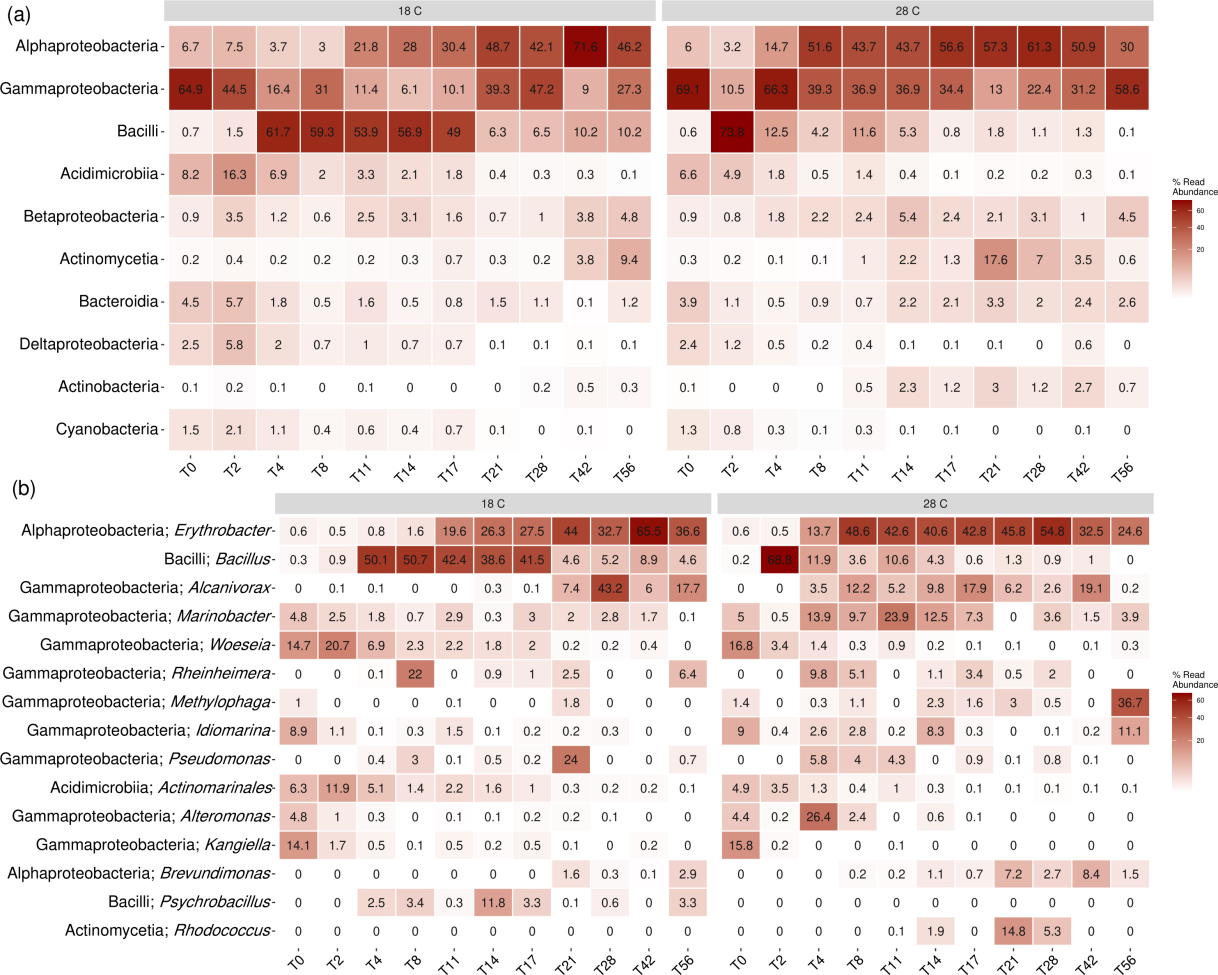
Microbial community evolution at the (**a**) class and (**b**) genus levels throughout the biodegradation of tar components at 18°C and 28°C.

The generated zOTUs could be classified into 5 kingdoms, 20 phyla, 46 classes, 116 orders, 193 families, and 340 genera. Bacteria mostly dominated the microbial community under both treatments, comprising around 98.67% of the microbial community at 18°C and 99.69% at 28°C. This is consistent with results reported in previous studies identifying bacteria as the most active agents in the biodegradation of petroleum hydrocarbons in marine environments (28, 34, 35). Heatmaps showing the distribution of the most dominant microbial populations throughout the biodegradation experiments were plotted to visualize the evolution of the microbial communities at 18°C and 28°C at the class (Fig. 4a) and genus (Fig. 4b) levels. A heatmap showing the evolution of the most dominant phyla is also presented in the supplemental material (Fig. S1).

##### Background microbial community structure

Gammaproteobacteria dominated in the background community classes, constituting 64.9% and 69.1% at 18°C and 28°C, respectively. This class comprises important obligate and generalist hydrocarbon degraders (36). To a lesser extent, Alphaproteobacteria also appeared in the background community (6.7% and 6%), as well as Acidimicrobiia (8.2% and 6.6%).

The genera that dominated in the background microbial community were *Woeseia* (14.7% and 16.8%), *Kangiella* (14.1% and 15.8%), *Idiomarina* (8.9% and 9%), and *Marinobacter* (4.8% and 5%) from the Gammaproteobacteria class, and *Actinomarinales* (6.3% and 4.9%) from the Acidimicrobiia class. *Woeseia* is a novel genus of facultative anaerobes, possessing a range of genes for the degradation of BTEX compounds (benzene, toluene, ethylbenzene, and xylenes), as well as alkanes and PAHs (37, 38). *Kangiella* is a genus of extracellular protein degraders typically isolated from marine sediments and seawater, comprising the strain *Kangiella aquamarine,* which is considered an efficient petroleum alkane degrader (37, 39). *Idiomarina* has been reported to thrive in stressful marine environments contaminated with petroleum hydrocarbons. *Marinobacter* comprises important species known for their marine hydrocarbon degradation and has been reported in several microbial community analyses associated with petroleum hydrocarbon-contaminated environments and hydrocarbon biodegradation (34, 35, 40–44).

Analysis of the microbial community structure at day 0 suggests that the indigenous microbial populations found in the marine sediments used in the biodegradation experiments comprise important hydrocarbon degraders. This can be attributed to the previous exposure of the sediments to petroleum oil and tar contamination, enriching the microbial community with microbes that are capable of petroleum hydrocarbon degradation.

##### Microbial dynamics at 18°C

**Fig 5:**
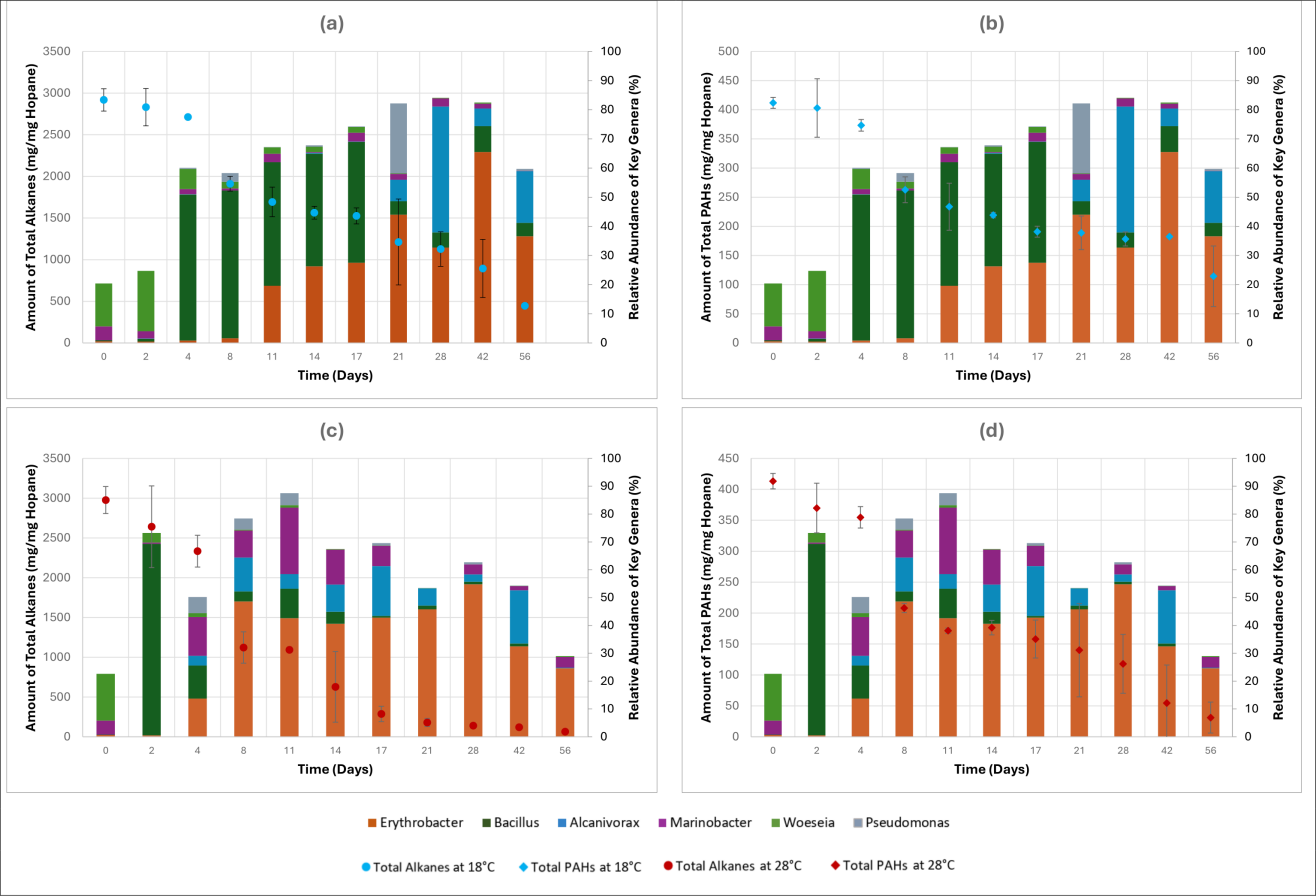
Evolution of key microbial genera in association with the degradation of (**a**) total alkanes at 18°C, (**b**) total PAHs at 18°C, (**c**) total alkanes at 28°C, and (**d**) total PAHs at 28°C.

Gammaproteobacteria, initially the most dominant microbial class, exhibited a decrease during the first 4 days of the lower temperature experiment, from 64.9% of the microbial community on day 0 to 16.4% on day 4. Despite this sharp decrease, important hydrocarbon degraders in this class of the genera *Marinobacter* and *Woeseia* were detected in considerable abundance during the first 4 days. Bacilli class showcased a dramatic increase, reaching 61.7% of the microbial community on day 4, starting from only 0.7% on day 0. Since the first 4 days of the 18°C experiment did not demonstrate significant biodegradation of total alkanes (Fig. 5a) or total PAHs (Fig. 5b), this may suggest that the microbial community was undergoing restructuring during this period to establish a consortium capable of degrading the hydrocarbon contaminants.

Bacilli continued to be the most dominant class between days 4 and 17 of the experiment, decreasing gradually to reach a relative abundance of 49% on day 17, represented mainly by the genus *Bacillus*. A gradual increase in the relative abundance of Alphaproteobacteria was observed during this period, from 3.7% on day 4 to 30.4% on day 17, mainly due to the increase in the abundance of the genus *Erythrobacter*. It is important to note that the period between days 4 and 17 exhibited the sharpest decrease in the amount of total alkanes and total PAHs, as shown in Fig. 5a and b. Bacterial strains in the *Bacillus* genus are known for their production of biosurfactants and are reported to have a role in the degradation of PAHs (45, 46). The increase in the relative abundance of members belonging to the Alphaproteobacteria class was reported in previous analyses of microbial communities during oil biodegradation (26, 47, 48).

Alphaproteobacteria continued to increase in abundance in the following period between days 21 and 56, reaching a maximum of 71.6% on day 42, mainly represented by the genus *Erythrobacter*. Gammaproteobacteria showcased an increase in relative abundance to reach a maximum of 47.2% on day 28, before decreasing to 27.3% on day 56. This was reflected by the increase in the genus *Alcanivorax* to reach a maximum of 43.2% in relative abundance on day 28 and the presence of the genus *Pseudomonas* with a relative abundance of 24% on day 21. *Alcanivorax* is an aerobic hydrocarbon degrader, particularly known for utilizing alkanes almost exclusively as sources of energy and carbon, and has been detected in high relative abundance during petroleum hydrocarbon degradation (41, 43, 48). It is interesting to note that high molecular weight (HMW) alkanes, particularly alkanes ranging in carbon number from nC22 to nC35, persisted during this study at 18°C and required the entire 56-day period to be degraded, as can be seen in Fig. 5a. The degradation of these alkanes occurred predominantly in the period between days 21 and 56, which coincides with the increase in the relative abundance of *Alcanivorax*. While *Alcanivorax* has been reported as a dominant hydrocarbon degrader in previous petroleum tar and weathered oil biodegradation studies (17, 49), the present study showcases the potential involvement of *Erythrobacter* and *Bacillus* in the biodegradation of petroleum tar hydrocarbons.

##### Microbial dynamics at 28°C

At the higher biodegradation temperature of 28°C, Alphaproteobacteria demonstrated a gradual steady increase in relative abundance from 6% on day 0 to a maximum of 61.3% on day 28, decreasing then to reach 30% on day 56. Similar to the lower temperature experiment, *Erythrobacter* was the major genus of this class. Gammaproteobacteria, on the other hand, showed a gradual decrease in relative abundance from 69.1% on day 0 to 22.4% on day 28, increasing after that to 58.6% on day 56. For the early sampling events (days 0–4), the results of the *t*-test did not show any statistically significant difference in microbial populations between the two temperatures. This is expected, as the microcosms for both treatments comprised beach sediments that were collected from the same site at the same time and were previously exposed to the same initial environmental conditions, leading them to have similar initial microbial communities, which was also observed in the NMDS plot of Fig. 3.

The Gammaproteobacteria class showcased major differences at 28°C compared to the 18°C experiment, as three main genera dominated throughout the duration of the experiment. *Alcanivorax* (*P* = 0.015), which was detected during the 18°C experiment only after day 21, was dominant throughout most of the 28°C experiment, starting from a relative abundance of 3.5% on day 4 to 19.1% on day 42. Similarly, *Marinobacter* (*P* = 0.033), which was detected in low relative abundance at 18°C, was a key player throughout the entire duration of the 28°C experiment, with a maximum relative abundance of 23.9% on day 11. It is important to note that both *Alcanivorax* and *Marinobacter* are important hydrocarbon degraders, with a special focus on straight-chain and branched alkanes (43). This can potentially explain the rapid decrease in the concentration of total alkanes between days 4 and 21 (Fig. 5c) when both genera were most abundant.

*Pseudomonas* was also detected between days 4 and 11, with a relative abundance between 4.3% and 5.8%. *Rhodococcus*, a genus in the class Actinomycetia that was completely undetected during the 18°C experiment, appeared between days 14 and 28, with a maximum relative abundance of 14.8% on day 21. The presence of *Pseudomonas* and *Rhodococcus* may be linked to the sharp decrease in total PAHs concentration during the 28°C biodegradation experiment observed between days 4 and 21 (Fig. 5d), as members of these two genera have been reported to be important degraders of PAHs (26, 43).

Bacilli*,* one of the most dominant classes at 18°C, was not significantly detected at 28°C. Except for a high relative abundance of 73.8% on day 2, Bacilli fluctuated between 0.8% and 12.5% throughout the 28°C experiment, mainly represented through the genus *Bacillus* (*P* = 0.015). The considerable decrease in the relative abundance of Bacilli under higher temperature conditions can be attributed to multiple factors. For instance, higher temperatures increase the toxicity of certain petroleum hydrocarbons, particularly PAHs which are a common substrate of *Bacillus*, and alter the properties of the contaminants, affecting their bioavailability (8, 50, 51). Another possible explanation for this decrease in abundance is the increased detection of other bacterial species at 28°C that are capable of degrading alkanes and PAHs, like *Pseudomonas* and *Rhodococcus*. Since both are important degraders of PAHs and alkanes, common substrates targeted by *Bacillus*, competition for resources might have led to the decrease in the relative abundance of *Bacillus*.

### Conclusion

This study assessed the potential of beach sediments collected from the coast of Lebanon to bioremediate tar contaminants that result from the weathering of petroleum oil following a spill event. It was concluded that the indigenous microbial populations were capable of degrading tar constituents, namely alkanes and PAHs, with a reported biodegradation rate of 0.0353 ± 0.0030 day^−1^ and 0.1106 ± 0.0083 day^−1^ for total alkanes at 18°C and 28°C, respectively, and 0.0238 ± 0.0041 day^−1^ and 0.0556 ± 0.0051 day^−1^ for total PAHs at 18°C and 28°C, respectively. The general trend of biodegradation observed was consistent with typical biodegradation behavior, with LMW compounds being degraded earlier and at faster rates than their HMW counterparts. In addition, the microbial community associated with the degradation of the tar components was studied, and the evolution of the involved microbial populations at the two biodegradation temperatures was monitored. The results indicated that the indigenous microbial community comprised important hydrocarbon degraders, with an evident dominance of members of the Gammaproteobacteria and Alphaproteobacteria classes, in addition to lower relative abundances detected for members of the Acidimicrobia, Deltaproteobacteria, and Cyanobacteria classes. As the biodegradation experiments progressed, successive restructuring of the microbial community was observed, favoring the dominance of different microbial populations specialized in the degradation of different tar components at specific stages of the biodegradation process and at defined temperatures, reflecting the progressive degradation of alkanes and PAHs. Microbial community analysis also showed the contribution of new genera to the biodegradation of tar hydrocarbons, such as *Bacillus*, as well as other genera that have been previously reported as key tar hydrocarbon degraders, including *Alcanivorax*, *Pseudomonas*, and *Marinobacter*. The findings of this study provide valuable insights for spill responders in the case of a future potential tar spill. By identifying microbial taxa associated with different stages of tar degradation, responders can use molecular or sequencing-based monitoring to assess whether indigenous microbial communities in a contaminated site have the potential to degrade tar naturally. If native microbial communities show limited capacity for tar degradation, the study’s findings on specialized degraders can inform the selection of microbial strains for bioaugmentation. In addition, understanding tar biodegradation kinetics and microbial succession patterns allows responders to predict degradation timelines and adjust remediation strategies accordingly.

## Data Availability

The sequence reads can be obtained from the National Center for Biotechnology Information (NCBI) using the accession number PRJNA1243352.

## SUPPLEMENTAL MATERIAL

Graphical abstract aem.00258-25-s0001.pdf Visual schematic of the study.

Highlights aem.00258-25-s0002.pdf Key points of the study.

Supplemental material aem.00258-25-s0003.pdf Data on chemical and bacteriological characterization of beach sediments, petroleum tar composition, DNA amplification, sequencing, and analysis, and microbial community diversity and evolution.

ASM does not own the copyrights to Supplemental Material that may be linked to, or accessed through, an article. The authors have granted ASM a non-exclusive, world-wide license to publish the Supplemental Material files. Please contact the corresponding author directly for reuse.
