# Supplementary material for: Biodegradation of petroleum tar in contaminated sediments of the Eastern Mediterranean shores and associated microbial dynamics
Source: Appl Environ Microbiol. 2025 Jun 12;91(7):e00258-25. doi: 10.1128/aem.00258-25 (PMC12285258; doi:10.1128/aem.00258-25)
Supplement: Graphical abstract — Visual schematic of the study. [file aem.00258-25-s0001.pdf]

Biodegradation Kinetics of Petroleum Tar in Marine Environments and Associated Microbial Dynamics

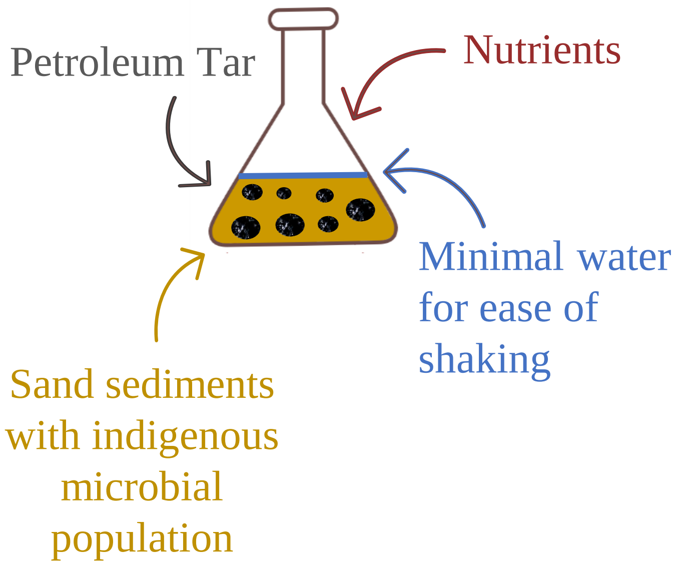

18°C and 28°C

56 Days

200 rpm

Depletion of Tar Hydrocarbons

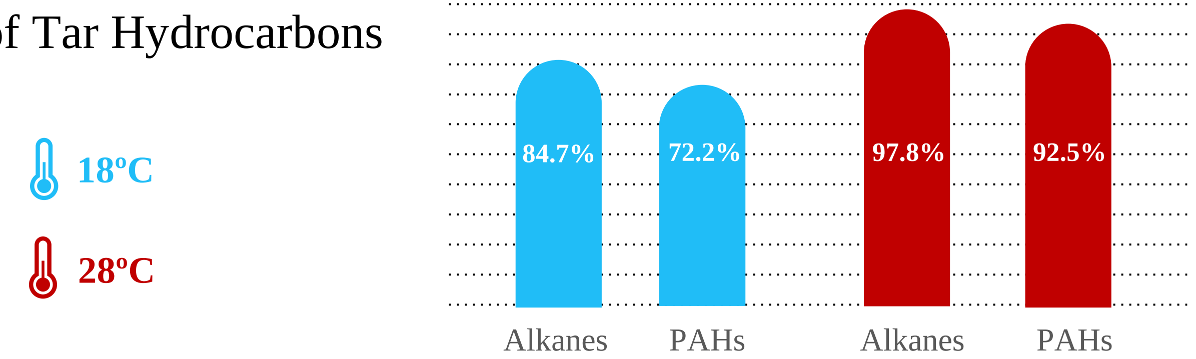

Evolution of Microbial Population

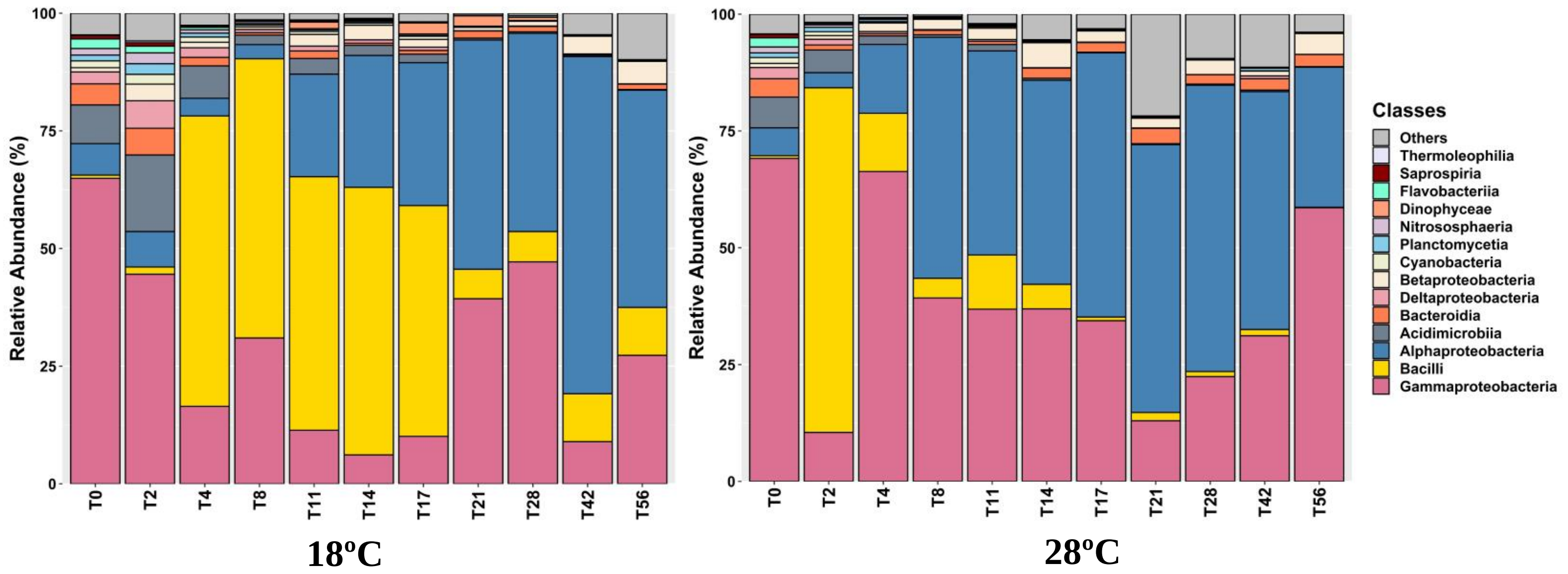

Al Haj Chehadeh, B., Ali Ahmad, F., Salam, D. A. (2024)
