# Supplementary material for: Biodegradation of petroleum tar in contaminated sediments of the Eastern Mediterranean shores and associated microbial dynamics
Source: Appl Environ Microbiol. 2025 Jun 12;91(7):e00258-25. doi: 10.1128/aem.00258-25 (PMC12285258; doi:10.1128/aem.00258-25)
Supplement: Highlights — Key points of the study. [file aem.00258-25-s0002.pdf]

Biodegradation of Petroleum Tar in Contaminated Sediments of the Eastern Mediterranean Shores and Associated Microbial Dynamics

Baraa Al Haj Chehadeh, Farah Ali Ahmad, Darine A. Salam*

Department of Civil and Environmental Engineering, Maroun Semaan Faculty of Engineering and Architecture, American University of Beirut, Beirut, Lebanon.

*Corresponding Author: Darine A. Salam. American University of Beirut, Maroun Semaan Faculty of Engineeering and Architecture, Munib and Angela Masri Bldg, M418. P.O.Box: 11-0236, Riad El Solh 1107 2020. Beirut, Lebanon. Email: ds40@aub.edu.lb, Phone: +961-1-350000-Ext: 3609, Fax: +961-1

Highlights

- Lower biodegradation rates measured for longer carbon chain alkanes and PAHs at 18°C.
- The background microbial community comprised generalist hydrocarbon degraders.
- The microbial community evolved during degradation to more specialized populations.
- Gammaproteobacteria and Alphaproteobacteria class members were key degraders.
- Erythrobacter and Bacillus were newly identified to be involved in tar degradation.
