# Supplementary material for: Biodegradation of petroleum tar in contaminated sediments of the Eastern Mediterranean shores and associated microbial dynamics
Source: Appl Environ Microbiol. 2025 Jun 12;91(7):e00258-25. doi: 10.1128/aem.00258-25 (PMC12285258; doi:10.1128/aem.00258-25)
Supplement: Supplemental material — Data on chemical and bacteriological characterization of beach sediments, petroleum tar composition, DNA amplification, sequencing, and analysis, and microbial community diversity and evolution. [file aem.00258-25-s0003.pdf]

Biodegradation of Petroleum Tar in Contaminated Sediments of the Eastern Mediterranean Shores and Associated Microbial Dynamics

Baraa Al Haj Chehadeh, Farah Ali Ahmad, Darine A. Salam*

Department of Civil and Environmental Engineering, Maroun Semaan Faculty of Engineering and Architecture, American University of Beirut, Beirut, Lebanon.

*Corresponding Author: Darine A. Salam. American University of Beirut, Maroun Semaan Faculty of Engineering and Architecture, Munib and Angela Masri Bldg, M418. P.O.Box: 11-0236, Riad El Solh 1107 2020. Beirut, Lebanon. Email: ds40@aub.edu.lb, Phone: +961-1-350000-Ext: 3609, Fax: +961-1

1. Petroleum Tar Chromatogram

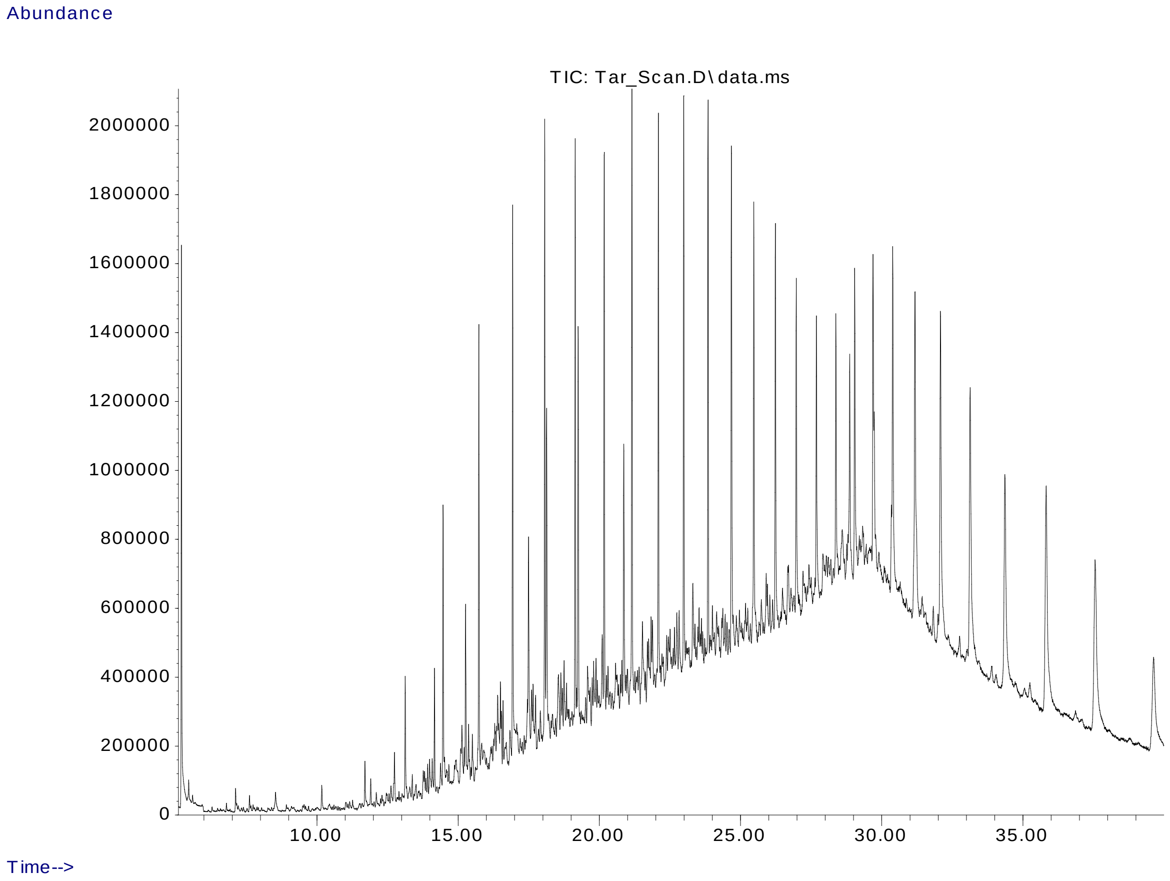

Figure S1: Chromatogram of tar sample collected from the August 2021 spill from Tyre Beach, Southern Lebanon

2. Chemical and bacteriological characterization of beach sediments

2.1 Chemical characterization

Background concentrations of nitrogen and phosphorous necessary for microbial activity were determined by testing the extracts of sieved sediment samples for nitrates-N, nitrites-N, ammonia-N, Total kjeldahl nitrogen, phosphate-P, and total phosphorous using spectrophotometry. The chemical compounds were extracted from the samples by mixing 30 g (wet weight) of beach sediment with 100 mL of distilled water at 300 rpm for 1 hour. Background nutrient levels in the sediment samples collected from the beach of Tyre are presented in Table .

Table S1: Background nutrient levels in Tyre beach sediments

	**Nitrogen Content (mg/Kg)**				**Phosphorus Content (mg/Kg)**	
**Nutrient**	Nitrate-N + Nitrite-N	Ammonia-N	Total Kjeldahl Nitrogen-N	Total Nitrogen-N	Reactive Phosphorus-P	Total Phosphorus
**Average**	0.31	0.11	0.10	0.57	BDL*	BDL
**SD**	0.01	0.02	0.04	0.02	-	-

***Below Detection Limit**

The sediment samples tested demonstrated measurable concentrations of total nitrogen (0.57 mg/Kg) but phosphorus content was below the detection limit. The measurable concentrations of nitrogen can be traced to the discharge of untreated sewage into seawater as well as the proximity of farmlands to the beach in Tyre. Despite the measurable concentrations of nitrogen in the sediment samples, the present concentrations do not satisfy the 100:5:1 C:N:P ratio needed for optimal biodegradation rates of petroleum hydrocarbons.

2.2 Bacteriological analysis

For the microbiological analysis of the sediments, triplicate sediment samples were processed for most probable number (MPN) analysis of alkane- and PAH-degrading bacteria according to the procedure outlined in Sakaya et al. (2019) (1). For each sample, approximately 10 g (wet weight) of beach sediment were added to 90 mL of a sterile detachment solution

containing 1 g/L disodium pyrophosphate ($Na_2H_2P_2O_7$) and 20 g/L of sodium chloride (NaCl), and mixed at 300 rpm for 1 hour. The extract underwent serial 10-fold dilutions in 96-well microtiter MPN plates. In each well, 175 µL of *Bushnell Haas* was used as a growth medium and 2 µL of light Arabian petroleum crude oil were added as a carbon source for the bacteria. Serial dilutions were performed by successively transferring 20 µL of the mixed content of a well to the subsequent well in the next row, starting with the undiluted extract in the first row. The plates were incubated at 20 °C for 14 days, and positive results were identified through the observation of oil emulsification in the wells, as compared to control wells where no sediment extract was added.

3. DNA amplification, sequencing, and analysis

3.1 DNA amplification and sequencing

DNA amplification and sequencing were conducted at MR DNA (Shalloowater, TX, USA). The extracted DNA was used as a template for polymerase chain reaction (PCR), whereby the V4 variable region of the 16S rRNA gene was amplified using the 515/806 primers, with barcode on the forward primer. The HotStarTaq Plus Master Mix Kit (Qiagen, USA) was used for 30 PCR cycles, and the amplification process was performed under the following conditions: 95 °C for 5 minutes, followed by 30 cycles at 95 °C for 30 seconds, 53 °C for 40 seconds, and 72 °C for 1 minute. A final elongation step was performed for 10 minutes at 72 °C. Following amplification, the obtained PCR products were checked in 2% agarose gel to evaluate the success of the amplification process and determine the relative intensity of bands.

Using unique dual indices, equal proportions of the samples were pooled together to multiplex the samples based on their DNA concentrations and molecular weight, after which the samples were purified using calibrated Ampure XP Beads. An Illumina DNA Library was then prepared using the resulting purified PCR product. A Miseq was used to perform sequencing according to the manufacturer's instructions, followed by the processing of the sequence data through MR DNA ribosomal and functional gene analysis pipeline. The analysis pipeline comprised joining the sequences as well as removing sequences with less than 150 base pairs (bp) and/or ambiguous base calls. The obtained sequences were filtered to improve their quality by setting a maximum expected error threshold of 1.0, after which they were dereplicated. The

resulting unique sequences were then denoised, whereby the sequences attributed to sequencing and/or PCR point errors were removed. Finally, chimeras were removed to obtain the final zero-radius operational taxonomic units (zOTUs), which were taxonomically classified using BLASTn against a curated database derived from the National Center for Biotechnology Information (NCBI) (www.ncbi.nlm.nih.gov) and compiled into both “counts” and “percentages” files at each taxonomic level. zOTUs are highly refined groupings, as zOTUs correspond to sequences that are exactly identical to one another, ensuring exact sequence matching for clustering. In the case of zOTUs, all correct biological sequences are identified, distinguishing sequences with even a single difference.

3.2 Statistical Analysis

Heatmaps were plotted to visualize the relative abundances of microbial communities at the phylum, class, and genus levels. In addition, a non-metric multidimensional scaling (NMDS) plot was derived based on the Bray-Curtis distance measure and according to the Hellinger transformation to visualize the relatedness and evolution of the microbial populations throughout the duration of the biodegradation experiments. Moreover, alpha diversity of the microbial populations was evaluated by calculating the Shannon diversity index.

4. Microbial community diversity and evolution

4.1 Microbial alpha diversity

Table S2: Shannon diversity indices of microbial populations in the sediment samples

Time	Temperature	Reads	Shannon
**T0**	18 °C	22548	5.060493
**T0**	28 °C	22465	4.948235
**T2**	18 °C	21325	5.726086
**T2**	28 °C	27001	4.060483
**T4**	18 °C	25824	4.874022
**T4**	28 °C	26085	3.820222
**T8**	18 °C	28115	3.870986
**T8**	28 °C	30090	3.620861
**T11**	18 °C	29409	4.024479
**T11**	28 °C	30715	3.45768
**T14**	18 °C	29485	3.884861
**T14**	28 °C	28786	3.935479

Time	Temperature	Reads	Shannon
**T17**	18 °C	29293	3.923295
**T17**	28 °C	29866	3.82512
**T21**	18 °C	29782	3.422604
**T21**	28 °C	29912	3.740674
**T28**	18 °C	29921	2.667964
**T28**	28 °C	30600	3.733964
**T42**	18 °C	31966	2.681785
**T42**	28 °C	29475	3.979906
**T56**	18 °C	29772	3.61689
**T56**	28 °C	29558	3.337613

T0 to T56 correspond to the 11 sampling events conducted on days 0, 2, 4, 8, 11, 14, 17, 21, 28, 42, and 56.

4.2 Microbial community evolution at the phylum level

Figure S1 presents the heatmap showing the evolution of the microbial communities during the tar biodegradation experiments at 18°C and 28°C.

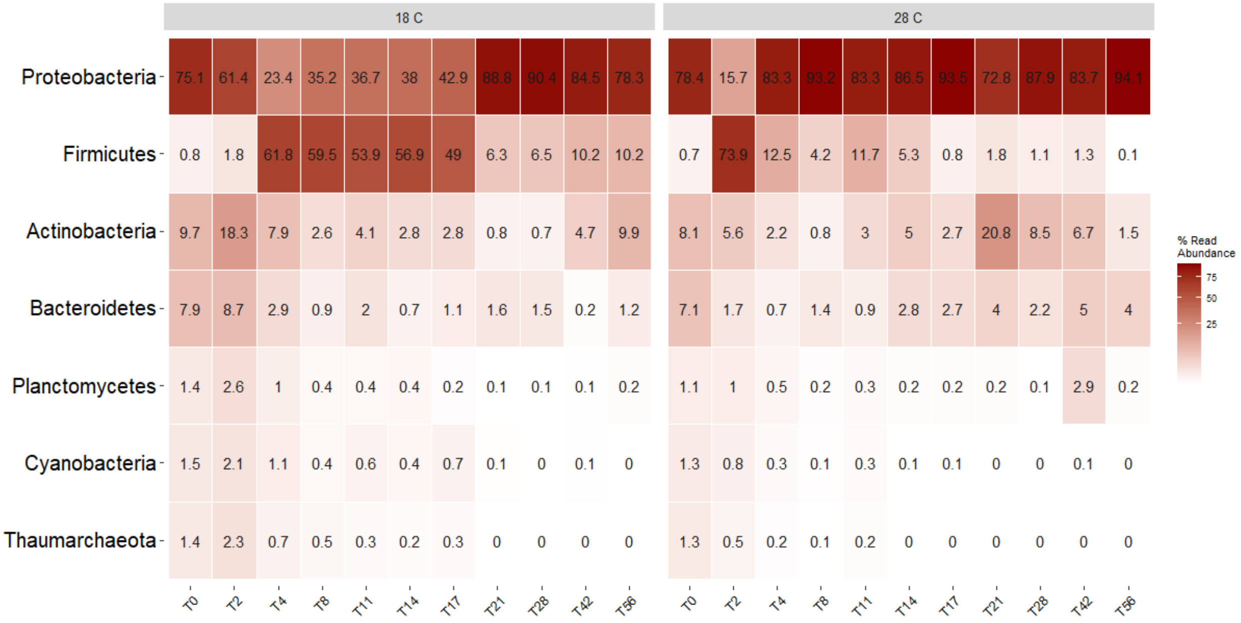

Figure S2: Microbial community evolution at the phylum level throughout the biodegradation of tar components at 18ºC and 28ºC

The phylum Proteobacteria dominated among the background community phyla in both treatments, constituting 75.1% and 78.4% of the microbial community on Day 0 at 18 °C and 28 °C, respectively. Actinobacteria (9.7% and 8.1%) constituted the second most dominant phylum

in the background community, followed by Bacteriodetes (7.9% and 8.1%), Planctomycetes (1.4% and 1.1%), Cyanobacteria (1.5% and 1.3%), and Thaumarchaeota (1.4% and 1.3%) in less significant proportions. Proteobacteria, along with Actinobacteria and Cyanobacteria are reported to be involved in hydrocarbon degradation (2– 4). In addition, previous studies indicate that Planctomycetes and Bacteriodetes might play a role in the degradation of PAHs (5, 6).

References

1. Sakaya K, Salam DA, Campo P. 2019. Assessment of crude oil bioremediation potential of seawater and sediments from the shore of Lebanon in laboratory microcosms. Sci Total Environ 660:227–235.
2. Das N, Chandran P. 2011. Microbial Degradation of Petroleum Hydrocarbon Contaminants: An Overview. Biotechnol Res Int 2011:1–13.
3. Sun Y, Wang H, Li J, Wang B, Qi C, Hu X. 2018. Nutrient-enhanced n-alkanes biodegradation and succession of bacterial communities. J Oceanol Limnol 36:1294–1303.
4. Zhou Z, Tran PQ, Kieft K, Anantharaman, K. 2020. Genome diversification in globally distributed novel marine Proteobacteria is linked to environmental adaptation. The ISME Journal 14(8): 2060–2077
5. Hilyard EJ, Jones-Meehan JM, Spargo BJ, Hill RT. 2008. Enrichment, isolation, and phylogenetic identification of polycyclic aromatic hydrocarbon-degrading bacteria from Elizabeth River sediments. Appl. Environ. Microbiol. 74(4): 1176–1182.
6. McGenity TJ, Folwell BD, McKew BA, Sanni GO. 2012. Marine crude-oil biodegradation: a central role for interspecies interactions. Aquat Biosyst 8.
